# Advance Care Planning Among People from Ethnic Minority and Non-Ethnic Minority Backgrounds with Poor-Prognosis Cancers: A Single-Site Retrospective Medical Record Review Study

**DOI:** 10.3390/healthcare14193235

**Published:** 2026-10-01

**Authors:** Ashfaq Chauhan, Ramya Walsan, Davinia Seah, Frances Bellemore, Ursula M. Sansom-Daly, Reema Harrison

**Affiliations:** 1 Australian Institute of Health Innovation, Macquarie University, Sydney, NSW 2019, Australia; 2 Sacred Heart Health Service, Sydney, NSW 2010, Australia; 3 IMPACCT-Improving Palliative, Aged and Chronic Care Through Clinical Research and Translation, Faculty of Health, University of Technology Sydney, Sydney, NSW 2007, Australia; 4 St Vincent’s Clinical School, University of New South Wales, Sydney, NSW 2010, Australia; 5 Faculty of Medicine, University of Notre Dame Australia, Darlinghurst Campus, Sydney, NSW 2010, Australia; 6 Behavioural Sciences Unit, School of Clinical Medicine, Discipline of Paediatrics and Child Health, Randwick Clinical Campus, UNSW Medicine and Health, UNSW Sydney, Randwick, NSW 2031, Australia; 7 Kids Cancer Centre, Sydney Children’s Hospital, Sydney, NSW 2031, Australia; 8 Sydney Youth Cancer Service, Nelune Comprehensive Cancer Centre, Prince of Wales Hospital, Sydney, NSW 2031, Australia; 9 Ingham Institute for Applied Medical Research, Liverpool, Sydney, NSW 2170, Australia

**Keywords:** advance care planning, cancer, ethnic minorities, culturally and linguistically diverse, end-of-life care, health equity

## Abstract

**Background/Objectives:** Studies indicate inequity in uptake of advance care planning (ACP) for some groups of people from ethnic minority backgrounds. There is limited evidence of disparity in the uptake of ACP among people from ethnic minority and non-ethnic minority backgrounds experiencing cancer in Australia. This exploratory study aimed to fill this gap. **Methods:** A single-site retrospective medical record review study was conducted. Random sampling was employed to extract data from eligible medical records of patients from ethnic minority and non-ethnic minority backgrounds with a poor-prognosis cancer diagnosis. **Results:** A total of 102 records were reviewed: 52 patients from ethnic minority backgrounds and 50 from the general population. Any ACP communication was documented in 77 medical records, with no statistically significant difference between groups (*χ*^2^ = 0.12, *p* = 0.732). A total of 326 ACP communications were documented. ACP communication rates were lower among patients from ethnic minority backgrounds, across total, formal, and informal communications, although none of the differences were statistically significant. Most ACP communications occurred in the palliative care setting (193/326); only six instances of ACP were about religious or cultural preferences for care. More patients from ethnic minority backgrounds died in acute care hospitals (12/16) when compared to the general population cohort (4/16), with discordance observed between preferred and actual place of death. **Conclusions:** This descriptive exploratory study found that most patient records reviewed contained at least one documented ACP communication. Whilst the rate of ACP was lower among people from ethnic minority backgrounds when compared to those from the general population, this difference was not statistically significant. Informal ACP communication was documented less frequently compared to formal ACP. Co-designed training and resources may assist cancer care clinicians in initiating timely and culturally appropriate ACP communication. Early referral to palliative care is recommended.

## 1. Introduction

Advance care planning (ACP) is described as a process of ongoing communication between a person, their families, or carers, and healthcare providers to ensure care is aligned with their preferences, wishes, and values [1]. Through ACP, a person would ideally discuss, document, and revise their care preferences throughout their care journey, formally or informally [2,3]. Formal documentation may occur through completion of legal or designated documents, such as medical intervention forms, during hospitalisations or care procedures; for example, a cardiopulmonary resuscitation (CPR) form, Advance Care Directive, or an Ambulance Care Plan [4]. Communication about future care may also occur informally through discussion between the patient, their loved ones, and health care providers, which may or may not be documented in medical or clinical notes [4]. This informal ACP may include discussions about preferred place of care at the end of life or death, or religious or cultural preferences of care that could not be captured in formal documents [4]. Informal ACP provides valuable information to health care providers and loved ones to enable care that is responsive to an individual’s needs and wishes for delivering person-centred end-of-life care [4].

In Australia, discussion and documentation of both formal and informal ACP has been promoted at a population level by local, state, and national initiatives, with the aim of improving alignment between a person’s needs, wishes, and the health care provided [1,5]. Advance Care Planning Australia is a federal government initiative that aims to promote and improve ACP for all Australians. A 2025 national survey of 3390 adults, led by Advance Care Planning Australia, highlighted that only one-third of Australians have engaged in some form of ACP, with the majority (19%) reporting discussing their future care with family or friends [6]. Only 6% reported completing a formal Advance Care Directive, and 13% appointed a substitute decision maker [6]. This survey noted that informal ACP conversations occurred between family members without a healthcare provider. Lack of documentation of these communications may result in healthcare delivery that may not align with a person’s wishes or preferences.

International evidence indicates that people from ethnic minority backgrounds experience inequity in uptake of ACP, but these studies have mainly focused on formal ACP communications. A 2023 systematic review identified 12 studies that documented formal ACP communications were present more commonly in the medical records of patients from non-ethnic minority backgrounds when compared to those from ethnic minority backgrounds [7]. People from ethnic minority backgrounds also experience poor quality of end-of-life care, which could be due to poor uptake of ACP. A 2023 systematic review of 29 studies found that people from ethnic minority backgrounds received more intensive care, had less hospice care, were more likely to die in a hospital, were less likely to engage in advance care planning, and were less likely to have good quality of care at the end-of-life [8]. In the Australian context, people from ethnic minority backgrounds include those born overseas, who have one or more parents born overseas, or who speak a language other than English at home, also referred to as culturally and linguistically diverse populations [9]. Broader Australian evidence exploring disparity in uptake of ACP among people from ethnic minority and non-ethnic minority backgrounds has also focused on formal ACP communications. A large prospective audit study of 4187 records of adults aged 65 years and over showed that there was a lower prevalence of completed Advance Care Directives among people from ethnic minority backgrounds (21.9%) compared to the non-ethnic minority population (28.9%) (*p* < 0.001) [10].

Cancer can be a life-limiting illness. ACP is particularly important for people affected by cancer as disease progression and prognosis vary [11]. Documentation of both formal and informal ACP communications becomes vital for care delivery during a period of uncertainties associated with cancer progression and prognosis. However, Australian studies examining ACP uptake among people with cancer have mainly focused on formal ACP documentation [10,12]. A single-site retrospective medical record review of 320 patients with advanced cancer identified lower odds of completing an Advance Care Directive for patients who received chemotherapy in the last two weeks of dying [12]. This study focused on only one type of formal ACP communication and did not examine differences in presence of ACP documentation between patients from ethnic minority and non-ethnic minority backgrounds. Another 2025 single-site retrospective medical record review study of 170 records examined documentation of any ACP communication among patients with metastatic solid organ malignancies enrolled in clinical trials [13]. Whilst this study reported no significant difference in any ACP documentation in the medical records of patients from ethnic minority and non-ethnic minority backgrounds, description of the type of ACP documentation (formal or informal) and the frequency of ACP communication was not provided [13].

Whilst people were more likely to have informal ACP conversations with their families or loved ones [6], current studies had largely focused on examining the uptake of formal ACP in cancer care settings, often using a single type of ACP, such as an Advance Care Directive [12]. Informal ACP communication also plays an important role in the delivery of person-centric end-of-life care, but these types of communications have not been thoroughly examined in cancer care settings in Australia [12,13]. Recognising these gaps in knowledge, the primary objective of this study was to determine and compare the frequency and type of ACP communication (formal and informal) documented in the medical records of patients from ethnic minority and non-ethnic minority backgrounds with poor-prognosis cancers. Considering that more people from ethnic minority backgrounds die in acute care hospitals when compared to the general population [8], the secondary objective of this study was to examine the number of patients with poor-prognosis cancers who had died in acute care hospitals across the two groups and whether the place of death was aligned with their preferred wishes.

## 2. Methods

### 2.1. Study Design

A retrospective medical record review was conducted as a recognised method to explore ACP documentation [13] and is reported using the standardized reporting requirements for observational studies (STROBE) guidelines [14]. Supplementary File S1 provides reporting against the STROBE guidelines.

### 2.2. Ethics

This study was conducted in accordance with the Australian National Statement on Ethical Conduct in Human Research (2023) [15] approved by the South Western Sydney Local Health District Human Research Ethics Committee (reference number: 2023/ETH00216). Waiver of consent was approved by the ethics committee.

### 2.3. Setting

The study was conducted at one public health service in the state of New South Wales in Australia. Approximately 2.3 million (29.3%) of New South Wales residents are from ethnic minority backgrounds [16]. This public health service was based in a metropolitan region and provided comprehensive oncology and palliative care across inpatient and outpatient (including community) settings. It had over 350 inpatient beds and a dedicated palliative care centre with a 12-inpatient bed unit and a 24 h community and outpatient consultative service. The service also received palliative care referrals from other health services in New South Wales.

### 2.4. Eligibility

A medical record was eligible for inclusion if it belonged to individuals aged ≥18 years, with a documented diagnosis of poor-prognosis cancer and recorded first episode of care between 1 January 2017 and 31 December 2020 at the participating health service. The first episode of care was defined as being seen by a health professional at the participating health service for a cancer-related illness and/or management for the first time. Patient records that documented a first episode of care outside of 1 January 2017 to 31 December 2020 and those who were younger than 18 years of age were excluded.

Eligible medical records belonged to patients born in Australia and those identified as from ethnic minority backgrounds. People from ethnic minority backgrounds were identified if they had one or more of the following ethnic minority indicators present (country of birth other than Australia, language spoken at home other than English, preferred language other than English, or interpreter required) [17,18]. Medical records of patients born in New Zealand, the United Kingdom, Ireland, the United States of America, Canada, and South Africa were excluded from being of an ethnic minority background based on prior research due to cultural similarities in populations from these countries to the general Australian population [18].

Poor-prognosis cancers included cancers with a 5-year survival rate less than the survival rate of all cancers combined, as indicated by Cancer Australia National Cancer Control Indicators for the 2016–2020 period [19]. In the period 2016–2020, people diagnosed with reported cancers were 71.2% likely to survive for at least 5 years after being diagnosed compared with comparable members of the general population [19]. We included cancers as poor-prognosis cancers that had a 5-year survival rate of less than 71% in the period 2016–2020. Based on this criterion, poor-prognosis cancers included pancreatic (13% 5-year relative survival), unknown primary (13.2% 5-year relative survival), brain (23.4% 5-year relative survival), liver (24.2% 5-year relative survival), oesophageal (25.1% 5-year relative survival), lung (25.6% 5-year relative survival), ovarian (48.1% 5-year relative survival), and bladder (57.3% 5-year relative survival) cancers [19]. All other cancer types were excluded.

For inclusion, formal ACP communication was defined as any communication that was conducted using a designated form for documenting wishes and preferences for future care. Informal ACP was defined as communication that did not use any designated form but was documented in the progress/clinical notes in the patient’s medical record.

### 2.5. Data Sources

The health service used three different electronic medical record (EMR) systems along with the paper-based medical records. Two EMR systems were used to document general medicine, cancer, and other specialty inpatient admissions and outpatient consultations. A third EMR system was used to document community palliative care consultations. A paper-based record system was used for inpatients admitted to the palliative care unit. All progress/clinical notes, medical or clinical assessments or reviews, doctors’ correspondence, admissions forms, transfer of care documents, family conferences or correspondence notes documented in the three EMR systems and the paper-based record system were reviewed. Data sources for each eligible patient were reviewed for a follow-up period of 5 years (or until the data were available if less than 5 years or until death was recorded) from the date of their first episode of care.

### 2.6. Data Collection Instrument

A data collection instrument or tool (Supplementary File S2) was developed in collaboration with research team members with subject matter expertise and knowledge of the ACP care processes at the health service (RH, USD, DS, and FB). This data collection tool allowed the reviewer (AC) to extract data from included medical records for socio-demographic, clinical, and ACP communications-related information relevant to the study objectives in a standardised manner.

### 2.7. Sample

The minimum total sample size required to determine and compare the prevalence of ACP documentation between the two groups with 5% precision at a 95% confidence interval was estimated to be a total of 326 medical records, assuming a prevalence of approximately 30% in the general population. However, the process of identifying and extracting data from four EMR systems covering a follow-up period of five years required considerable time (approximately 2.5 h) and financial resources.

Data from three EMR systems were first reviewed, and following this, the paper-based notes had to be requested separately from the medical record department. These considerations were discussed with the research team, and the sample size was amended. Accordingly, a pragmatic final sample of 102 medical records (52 patients from ethnic minority backgrounds and 50 from the general population) was therefore considered sufficient for this exploratory study. Two additional records of patients from ethnic minority backgrounds were identified as eligible in the process of record identification and data collection. A decision was made to include these in the final analysis. The reduced sample was considered appropriate for this descriptive exploratory study, while recognizing that it would provide less precise prevalence estimates and limited ability to identify modest differences between groups. Therefore, between-group analyses were interpreted descriptively and should be regarded as exploratory.

### 2.8. Identification of Eligible Medical Records

A list of patients with a diagnosis of poor-prognosis cancers and with a potential first episode of care during the eligible timeframe (1 January 2017 to 31 December 2020) was compiled by the health service data administration department. This list contained a total of 3221 names and included information on medical record identifier, cancer diagnosis, country of birth, preferred language, and interpreter required status against each name. A random number generator was used to generate a random list of numbers from 1 to 3221. These numbers were then applied sequentially to the list of patients compiled by the data administration department. The eligibility of the corresponding patient records was checked across the EMRs, and those eligible were included for review and data extracted. Records of patients from ethnic minority backgrounds were identified using the four indicators as documented in the eligibility criteria. This process continued until the required number of eligible medical records were identified and included across the two groups.

### 2.9. Data Extraction

Data extraction occurred in batches of six, as paper records were required to be requested separately from the medical record department, which could only be done in small batches. Approximately one week was required by the medical record department to provide the paper-based records for the purpose of review. For each of the six medical records, data were first extracted from EMR systems by one reviewer (AC) using the standardised data extraction tool. Following this, data from paper-based medical records were extracted after they were provided by the medical records department. This process continued until data were extracted from all the medical records.

The process of data extraction, any issues arising during the process, and preliminary findings from the data extracted were discussed with the lead (RH) in fortnightly meetings. Any issues were resolved in these meetings. When further site-specific inputs were required, the site investigators were consulted (FB; DS) to resolve any issues identified. The wider research team was updated on the progress of data extraction in monthly project team meetings.

### 2.10. Measurements

#### 2.10.1. Outcome Variables

The main outcome variables for the study were the number and type of ACP communication documented in the medical records. Data on the presence of any ACP communication was extracted into two main categories (yes or no), and the number of ACP communications documented per medical record was recorded as a count variable. Where ACP was documented, each respective ACP communication was categorised as formal or informal, and the numbers of formal and informal ACP communications per medical record were also recorded as separate count variables. A separate entry was then made for each communication noting the date, type and setting of the communication. Supplementary File S2 provides the categories used to group the data.

The secondary outcome variable was the place of death. Data on place of death for the included medical records that had recorded death during the review period were categorised into five categories: acute care hospital, palliative or hospice care, patient’s home, residential aged care home, or other. This information was available as administrative data in EMR systems.

#### 2.10.2. Predictive Variables

Predictive variables comprised socio-demographic and clinical care indicators. Socio-demographic variables comprised age, sex, country of birth, preferred language, interpreter required, language spoken at home, religion, parents’ country of birth and time since arrival to Australia. Specific socio-demographic data were extracted from the medical records and then categorised to assist with data analysis. Data on age were collected as continuous data and then converted into three categories. Country of birth data were categorised into ‘Australia’ and ‘not-Australia’. Preferred language-related data were categorised into ‘English’, ‘non-English’, or ‘not available’. Sex (male/female) and interpreter required (yes/no) data were available as categories in the medical records and extracted accordingly. Missing data were marked as ‘not available’ for preferred language and interpreter required status and were not included in the analysis. Information relating to religion, language spoken at home, parent/s country of birth, refugee status or time since arrival in Australia was not consistently recorded and hence was not included in the analysis.

Clinical-care indicators included poor-prognosis cancer diagnosis, the presence of additional diagnosis alongside cancer, and whether the patient was referred to or seen by palliative care. Information on additional diagnosis was collected from the medical records and later categorised into two categories (yes or no) for the purpose of analysis. Referral to or consultation by palliative care was collected as categorical data (yes, no). As each medical record was reviewed for a period of five years, data on performance status and cancer stage were not collected. This was a methodological limitation of the study, as the comparison between medical records of patients from ethnic minority and non-ethnic minority patients who died in an acute care hospital could not be conducted.

### 2.11. Data Analysis

Data analysis was conducted using IBM SPSS (Version 31). Data analysis was guided by the two study objectives, available sample size, and type of data collected. Descriptive statistics, such as frequencies and proportions, were used to summarise and provide an overview of the data and associated variables. Categorical data for predictive and outcome variables were reported as frequencies and proportions.

Pearson’s chi-square test or Fisher’s exact test was used to compare the two groups for the presence of any ACP communication, any formal ACP communication, and any informal ACP communications. The numbers of total, formal, and informal ACP communications per medical record were treated as count outcomes. The distributions and dispersion of the count outcomes were assessed to determine the appropriate regression model. Dispersion statistics ranged from 1.52 to 2.01, indicating overdispersion across the count outcomes. Negative binomial regression was used to compare ACP communication rates between the two population groups. Population group was included as the explanatory variable, with the general population group as the reference category. Results were reported as incidence rate ratios (IRRs) with 95% confidence intervals. Detailed comparisons of the type and setting of individual ACP communications were not undertaken because of small numbers within several categories and because individual patients could contribute multiple ACP communications. These variables were therefore reported descriptively.

## 3. Results

**Figure 1:**
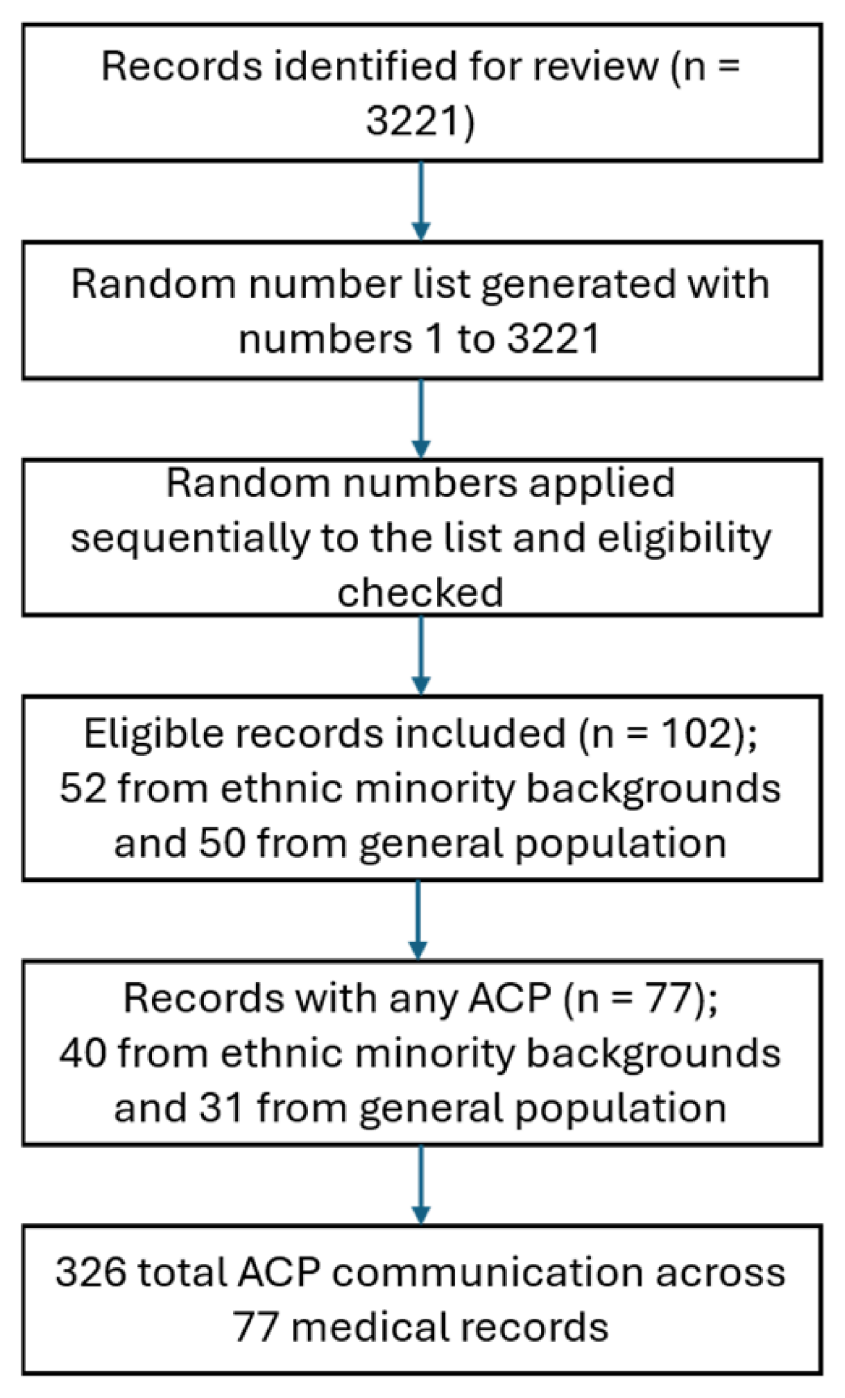
Flowchart describing medical record selection and number of records reviewed.

From the initial list of 3221 names, one hundred and two patient records were reviewed and included as the final sample. This comprised 52 (50.1%) patients from ethnic minority backgrounds (Figure 1).

The age of the patients ranged from 22 to 94 years, with a median age of 74 years (SD 12.38). Thirty-one different countries of birth, including Australia, were recorded. The top two non-Australian countries of birth were China (7/52) and Russia (5/52). Twenty-three records (44.2%) documented a non-English preferred language, while 15 records (28.8%) documented ‘interpreter required’ in the EMR. Three records did not document preferred language, and seven did not document interpreter required status and were excluded from analysis. No statistically significant differences in demographics and clinical care variables between the medical records of patients from ethnic minority backgrounds and those from the general population were observed (Table 1).

### 3.1. Frequency of ACP Communication

**Figure 2:**
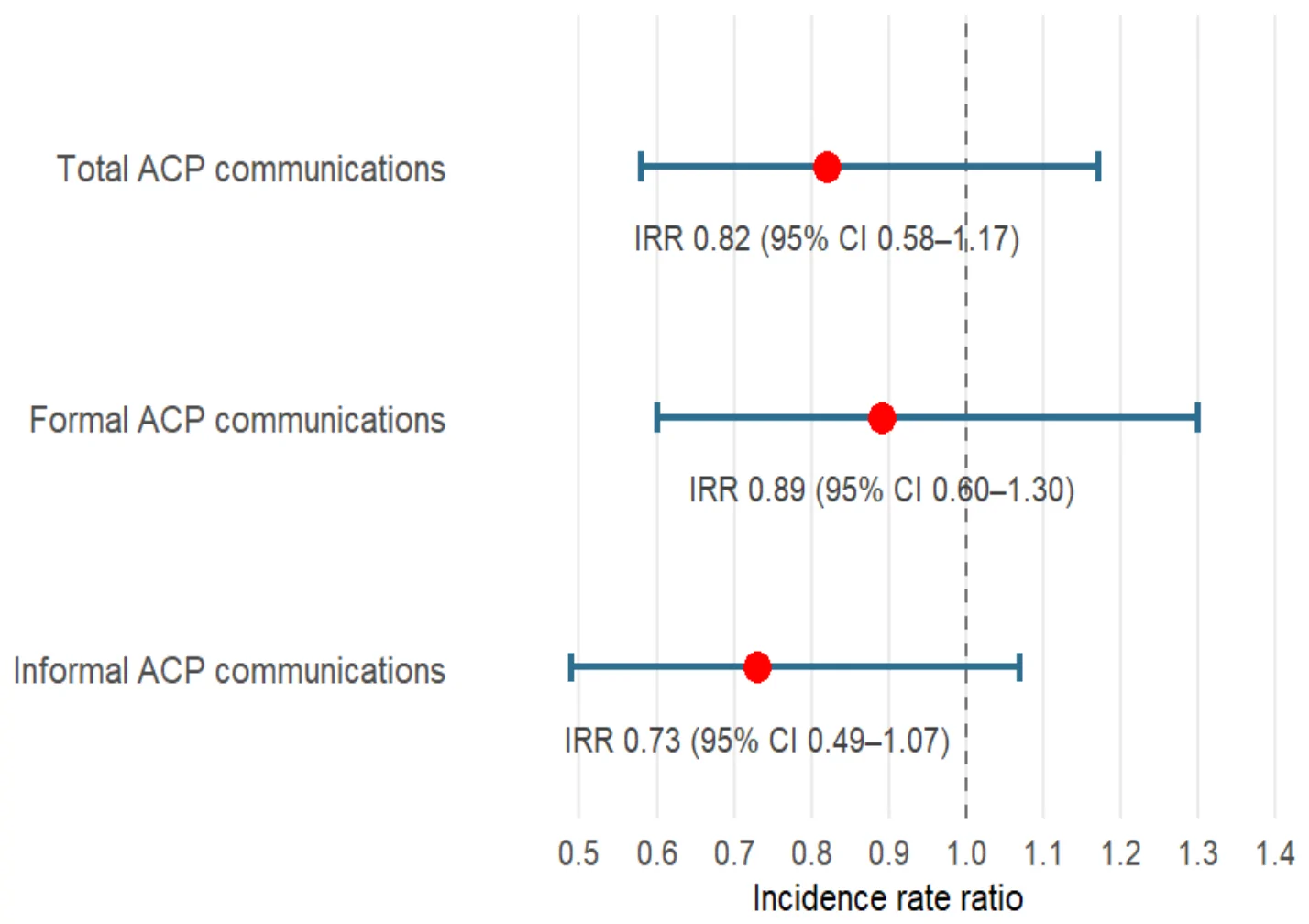
Incidence rate ratios for total, formal and informal ACP communications by population group (ethnic minority population compared with general population).

Any ACP communication (formal or informal) was documented in over 75% (77/102) of the medical records. No statistically significant difference was observed regarding the presence of any ACP communication between the two groups (Table 2). The number of ACP communications per record ranged from 0 to 10, with a mean of 3.2 (SD 2.55) and a median of three. A total of 326 ACP communications were documented, comprising 176 communications in the general population group and 150 in the ethnic minority group. The mean number of ACP communications per medical record was 3.52 (SD 2.73) in the general population group and 2.88 (SD 2.35) in the ethnic minority group. Negative binomial regression indicated a lower estimated rate of ACP communications in the ethnic minority group; however, the difference was not statistically significant (IRR 0.82, 95% CI 0.58–1.17, *p* = 0.269) (Figure 2).

For formal ACP communications, the mean number per medical record was 2.04 (SD 1.96) in the general population group and 1.81 (SD 1.66) in the ethnic minority group (IRR 0.89, 95% CI 0.60–1.30, *p* = 0.537). For informal ACP communications, the corresponding means were 1.48 (SD 1.30) and 1.08 (SD 1.19), respectively (IRR 0.73, 95% CI 0.49–1.07, *p* = 0.106). Although the estimated rates of total, formal, and informal ACP communications were consistently lower in the ethnic minority group, none of these differences reached statistical significance. (Figure 2) Any ACP communication was associated with palliative care team consultation (*χ*^2^ = 43.28, *p* < 0.001) and death being recorded in the medical records (*χ*^2^ = 64.42, *p* < 0.001).

### 3.2. Type and Setting of ACP Communication

The most common formal ACP communication was the presence of a CPR or a Patient Expectations of Care form (a local form created at the site to summarise Advance Care Directive, goals of care and Advance Care Plan). These forms were completed for patients when they were admitted to hospital or palliative care as part of routine care processes.

The most common informal communications were discussions about preferred place for end-of-life care or death and conversations about future treatment or procedures (Table 3). Of the total 326 ACP communications, only six were about religious and cultural preferences of care. Almost 60% of ACP communications documented occurred in palliative care settings, covering inpatient palliative care units and community palliative care.

### 3.3. Ethnicity and Place of Death in Acute Care

A total of 75 medical records documented that the patient had died: 36 (48%) of those from the general population and 39 (52%) of those from ethnic minority backgrounds. For 39 medical records of patients from ethnic minority backgrounds, 24 (61.5%) recorded deaths in a palliative care centre, 12 (30.8%) in an acute care hospital, and three (7.7%) at home or in a nursing home. Of the 36 medical records of patients from the general population, 25 (69.5%) recorded deaths in a palliative care centre, seven (19.4%) at home or in a nursing home, and four (11.1%) in an acute care hospital. More patients from ethnic minority backgrounds (75%, 12/16) died in acute care hospitals when compared to those from the general population (25%, 4/16).

Of the 16 records that documented death in an acute care hospital, informal communication about preferred place of death was not recorded in 12 records (eight belonging to patients from ethnic minority backgrounds). Four medical records of patients from ethnic minority backgrounds documented preferred place of death as a non-acute care hospital but recorded death in an acute care hospital.

## 4. Discussion

The present study, conducted in an Australian cancer setting, examined the frequency and type of ACP communication among patients from ethnic minority and non-ethnic minority backgrounds over a five-year follow-up period. Almost three-quarters of the patient records reviewed had at least one ACP communication documented. The mean number of ACP communications was lower among patients from ethnic minority backgrounds when compared to those from the general population (2.88 vs. 3.52). Analysis also showed lower estimated rates of total, formal, and informal ACP communications in the ethnic minority group compared with the general population, although these differences were not statistically significant. More patients from ethnic minority backgrounds died in acute care hospitals when compared to those from the general population, with the majority documenting no record of communication about preferred place of death. Given the small number of acute hospital deaths and the absence of adjustment for potential demographic and clinical confounders, this finding should be considered descriptive and cannot be attributed independently to ethnic background. Further studies with larger samples and appropriate adjustment for potential confounders are needed to confirm this finding.

The study indicated the presence of ACP communication in 75% of the medical records reviewed, demonstrating that the uptake of ACP was high when compared to recent studies where lower uptake had been documented [6]. However, these studies were conducted in different settings [6,10]. A 2020 prospective review study of 458 records of patients with cancer indicated that 30% of the records had any ACP documentation; however, no separate analysis was conducted for 105 records that belonged to patients from ethnic minority backgrounds [20]. This study reviewed medical records of older Australians across general practice, hospital, and residential aged care homes. A review of 170 medical records of cancer patients enrolled in clinical trials indicated the presence of any ACP in 64% (109/170) of medical records [13]. Increased documentation of ACP communication in our study could be due to existing care processes at the study site. As part of admission to the hospital or palliative care unit at the participating site, a CPR form or a Patient Expectations of Care form was completed for patients. This finding indicates that integration of formal ACP communication or document completions into existing care processes may result in increased uptake of ACP.

Findings from this study reported lower informal ACP communications documentation when compared to formal ACP for people from ethnic minority backgrounds. This novel finding may indicate an emphasis on formal ACP or a lack of care processes through which informal ACP communications were conducted and documented. Our study also documented that more patients from ethnic minority backgrounds (12/16) died in hospital as compared to those from the general population. It is likely that some patients from ethnic minority backgrounds may prefer to continue receiving care in hospital closer to death [21]. But our study identified that the preferred place of end-of-life care or death was not documented in many of the records of the patients who died in acute care hospitals. It is likely that these conversations may have occurred but were not documented, or that the clinicians may have lacked confidence in initiating these conversations [22]. Lack of skills of cancer clinicians in conducting or facilitating conversations about spirituality [23] may also reflect our findings that only 2% of documented ACP was about cultural and religious preferences of care. There are two practice implications to promote uptake of informal ACP. Firstly, integrating mechanisms through which informal ACP communications (such as preferred place of death) are conducted and documented as part of exiting care processes may result in delivery of goal-concordant care. Secondly, clinicians can be provided with training to build their confidence and skills in initiating or conducting informal ACP communication. Support from health services will also be needed to facilitate documentation [24]. In addition to training, culturally adapted resources and tools may also be used to facilitate ACP conversations about spirituality and cultural or religious preferences for care [25].

The study indicated that having a palliative care consult was significantly associated with the presence of any ACP communication. Findings highlighted that almost 60% of ACP communications occurred in a palliative care setting. Early referral to palliative care for cancer patients is an evidence-based recommendation promoted through nationally endorsed Optimal Care Pathway frameworks [26]. A recent qualitative study with Mandarin- and Cantonese-speaking people affected by cancer highlighted their desire to engage in ACP but noted that cancer specialists started ACP conversations or made referrals to other relevant staff to have these conversations at a late stage of disease progression [27]. Working with people from ethnic minority backgrounds affected by cancer, cancer clinicians, and other stakeholders, culturally appropriate referral pathways can be co-designed to improve timely access to palliative care.

Whilst the study attempted to collect diverse socio-cultural data, routine collection was limited to country of birth, preferred language indicators and interpreter requirement. Reliance solely on country of birth and preferred language to identify people from ethnic minority backgrounds may mean that medical records of other eligible patients may have been excluded [28]. Routine collection of a range of socio-cultural and ethnicity variables is recommended to capture ethnic and cultural diversity of populations accessing the Australian cancer care system [29]. Limited resources available to conduct a retrospective review were a challenge, especially when multiple electronic and paper-based medical record systems were used. A single digital patient record being implemented across public hospitals in New South Wales may address some of these challenges.

### Strengths and Limitations

The robust review of the medical records with a five-year follow-up period provides a comprehensive assessment of ACP communication documented in the patient records. Notes from multidisciplinary staff were reviewed, which was reflective of the fact that ACP communication may occur with any staff. This is also a first study in Australia that explored informal ACP communication. These are the main strengths of the study. The relatively small sample size remains an important limitation and reduced the precision of estimates and the ability to detect modest between-group differences. Although count-based analyses were undertaken for total, formal and informal ACP communications, detailed comparisons of specific communication types and settings were limited by small cell frequencies. The small number of acute hospital deaths also precluded reliable multivariable adjustment for potential demographic and clinical confounders. In addition, potentially relevant clinical variables, including cancer stage and performance status, were not collected. Accordingly, findings should be considered descriptive and cannot be attributed independently to ethnic background. As a medical record review study, the data collected were reliant on the documentation of ACP communication; likely not all the communication (especially informal) would have been documented in the medical records. Further, the quality and legibility of handwritten notes may have had an impact on study findings. The study site was based in a metropolitan area in the state of New South Wales in Australia and provided community and inpatient palliative care services. The findings may not be directly transferable to other healthcare services, settings, and countries.

## 5. Conclusions

This exploratory study provides evidence on the frequency and type of ACP communication documented in the medical records of patients from ethnic minority backgrounds and those of the general population. The study highlighted that the rate of ACP communication is lower among people from ethnic minority backgrounds affected by cancer when compared to those from non-ethnic minority backgrounds. Palliative care consultation is a facilitator for ACP uptake, but training is required for oncology clinicians to initiate timely ACP communication in cancer care settings and referral to the palliative care team. A co-designed methodology could be employed to develop resources with end-users and other relevant stakeholders to improve staff confidence in initiating and conducting ACP communication and making timely referral to palliative care for delivery of person-centred end-of-life care for people from ethnic minority backgrounds.

## Figures and Tables

**Table 1:** Socio-cultural demographic and clinical care description of the sample included in the study.

Variable	General Population	Ethnic Minority Populations	Total	*χ*^2^ (*p*, *n*)
Sex				0.61 (0.436, 102)
Male	25 (50%)	30 (57.7%)	55 (53.9%)	
Female	25 (50%)	22 (42.3%)	47 (46.1%)	
Age at the time of the first episode of care				3.95 (0.139, 102)
19–60	12 (24%)	5 (9.6%)	17 (16.7%)	
61–75	17 (34%)	23 (44.2%)	40 (39.2%)	
76 & +	21 (42%)	24 (46.2%)	45 (44.1%)	
Cancer type				1.16 (0.762, 102)
Lung	25 (50%)	23 (44.2%)	48 (47%)	
Pancreatic	6 (12%)	10 (19.2%)	16 (15.7%)	
Liver	6 (12%)	7 (13.5%)	13 (12.8%)	
Others *	13 (26%)	12 (23.1%)	25 (24.5%)	
Any other diagnosis				0.93 (0.334, 102)
Yes	42 (84%)	47 (90.4%)	89 (87.3%)	
No	8 (16%)	5 (9.6%)	13 (12.7%)	
Referred to palliative care				0.61 (0.433, 102)
Yes	34 (68%)	39 (75%)	73 (71.6%)	
No	16 (32%)	13 (25%)	29 (28.4%)	
Seen by palliative care staff				0.01 (0.925, 102)
Yes	36 (72%	37 (71.2%)	73 (71.6%)	
No	14 (28%)	15 (28.8%)	29 (28.4%)	
Death recorded in the medical records				0.19 (0.731, 102)
Yes	36 (72%)	39 (75%)	75 (73.5%)	
No	14 (28%)	13 (25%)	27 (26.5%)	

* Others included oesophageal, bladder, brain, ovarian, and unknown primary in situ cancers.

**Table 2:** Frequency of ACP communication among patients with poor-prognosis cancer by population group.

Variable	General Population	Ethnic Minority Populations	Total	*χ*^2^ (*p*, *n*)
Any ACP communication				0.12 (0.732, 102)
Yes	37 (74%)	40 (76.9%)	77 (75.5%)	
No	13 (26%)	12 (23.1%)	25 (24.5%)	
Any formal ACP communication				0.12 (0.79, 102)
Yes	34 (68%)	37 (71.2%)	71 (69.6%)	
No	16 (32%)	15 (28.8%)	31 (30.4%)	
Any informal ACP communication				0.47 (0.495, 102)
Yes	34 (68%)	32 (61.5%)	66 (64.7%)	
No	16 (32%)	20 (38.5%)	36 (36.3%)	
Total number of ACP communications per record, mean (SD)	3.52 (2.73)	2.88 (2.35)	3.19 (2.55)	IRR 0.82 (95% CI 0.58–1.17), *p* = 0.269
Formal ACP communications per record, mean (SD)	2.04 (1.96)	1.81 (1.66)	1.92 (1.81)	IRR 0.89 (95% CI 0.60–1.30), *p* = 0.537
Informal ACP communications per record, mean (SD)	1.48 (1.30)	1.08 (1.19)	1.28 (1.26)	IRR 0.73 (95% CI 0.49–1.07), *p* = 0.106

**Table 3:** Presence of any ACP and its association with socio-demographic and clinical-care indicators.

Variable	Formal ACP Documentation (*n* = 196)	Informal ACP Documentation (*n* = 130)	Total (*n* = 326)
Ethnic minority background			
Setting			
Inpatient palliative care unit	106 (54.1%)	25 (19.2%)	131 (40.2%)
Community palliative care	14 (7.1%)	48 (36.9%)	62 (19.0%)
Inpatient	49 (25%)	37 (28.5%)	86 (26.4%)
Outpatient	4 (2%)	17 (13.1%)	21 (6.4%)
Emergency Department	18 (9.2%)	-	18 (5.5%)
Nursing home	2 (1%)	3 (2.3%)	5 (1.5%)
Type of ACP communication			
Acute Resuscitation form	117 (59.7%)	-	117 (35.9%)
Patient expectations of care form	54 (27.6%)	-	54 (16.6%)
Preferred place of end-of-life care or death	-	37 (28.5%)	37 (11.3%)
Future treatment directives	2 (1.0%)	29 (22.3%)	31 (9.5%)
Ambulance care plan	14 (7.1%)	3 (2.3%)	17 (5.2%)
Substitute decision maker	4 (2.0%)	11 (8.5%)	15 (4.6%)
EoL or death, palliative sedation	-	14 (10.8%)	14 (4.3%)
Concerns and hopes for the future	-	7 (5.4%)	7 (2.1%)
Prognosis and life expectancy	-	6 (4.6%)	6 (1.8%)
Personal goals, wishes and wills	-	13 (10.0%)	13 (4.0%)
Religious and cultural preferences	-	6 (4.6%)	6 (1.8%)
Advance Care Plan	1 (0.5%)	-	1 (0.3%)
Advance Care Directive	3 (1.5%)	1 (0.8%)	4 (1.2%)

Statistical testing was not conducted for the setting and type of ACP communication due to small cell counts.

## Data Availability

The datasets generated during and/or analysed during the current study are available from the corresponding author upon reasonable request and with appropriate ethical approvals.

## Supplementary Materials

The following supporting information can be downloaded at https://www.mdpi.com/article/10.3390/healthcare14193235/s1: Supplementary File S1: STROBE Checklist; Supplementary File S2: Data extraction tool.
