# Supplementary material for: Advance Care Planning Among People from Ethnic Minority and Non-Ethnic Minority Backgrounds with Poor-Prognosis Cancers: A Single-Site Retrospective Medical Record Review Study
Source: Healthcare (Basel). 2026 Oct 1;14(19):3235. doi: 10.3390/healthcare14193235 (PMC13665487; doi:10.3390/healthcare14193235)
Supplement: Supplementary file 1 [file healthcare-14-03235-s001.zip › healthcare-4532529-SI.pdf]

Supplementary File S1: STROBE Checklist

STROBE Statement—Checklist of items that should be included in reports of ***cohort studies***

	Item No	Recommendation	Page No
**Title and abstract**	1	(*a*) Indicate the study's design with a commonly used term in the title or the abstract	1
		(*b*) Provide in the abstract an informative and balanced summary of what was done and what was found	1-2
**Introduction**			
Background/rationale	2	Explain the scientific background and rationale for the investigation being reported	2--3
Objectives	3	State specific objectives, including any prespecified hypotheses	3
**Methods**			
Study design	4	Present key elements of study design early in the paper	3
Setting	5	Describe the setting, locations, and relevant dates, including periods of recruitment, exposure, follow-up, and data collection	4-6
Participants	6	(*a*) Give the eligibility criteria, and the sources and methods of selection of participants. Describe methods of follow-up	4
		(*b*) For matched studies, give matching criteria and number of exposed and unexposed	4
Variables	7	Clearly define all outcomes, exposures, predictors, potential confounders, and effect modifiers. Give diagnostic criteria, if applicable	6
Data sources/ measurement	8*	For each variable of interest, give sources of data and details of methods of assessment (measurement). Describe comparability of assessment methods if there is more than one group	4-5
Bias	9	Describe any efforts to address potential sources of bias	5
Study size	10	Explain how the study size was arrived at	5-6
Quantitative variables	11	Explain how quantitative variables were handled in the analyses. If applicable, describe which groupings were chosen and why	6-7
Statistical methods	12	(*a*) Describe all statistical methods, including those used to control for confounding	6-7
		(*b*) Describe any methods used to examine subgroups and interactions	6-7
		(*c*) Explain how missing data were addressed	6-7
		(*d*) If applicable, explain how loss to follow-up was addressed	NA
		(*e*) Describe any sensitivity analyses	NA
**Results**			
Participants	13*	(a) Report numbers of individuals at each stage of study—eg numbers potentially eligible, examined for eligibility,	7

		confirmed eligible, included in the study, completing follow-up, and analysed	
		(b) Give reasons for non-participation at each stage	NA
		(c) Consider use of a flow diagram	7
Descriptive data	14*	(a) Give characteristics of study participants (eg demographic, clinical, social) and information on exposures and potential confounders	7-8
		(b) Indicate number of participants with missing data for each variable of interest	7-8
		(c) Summarise follow-up time (eg, average and total amount)	NA
Outcome data	15*	Report numbers of outcome events or summary measures over time	8-9
Main results	16	(*a*) Give unadjusted estimates and, if applicable, confounder-adjusted estimates and their precision (eg, 95% confidence interval). Make clear which confounders were adjusted for and why they were included	8-10
		(*b*) Report category boundaries when continuous variables were categorized	8
		(*c*) If relevant, consider translating estimates of relative risk into absolute risk for a meaningful time period	NA
Other analyses	17	Report other analyses done—eg analyses of subgroups and interactions, and sensitivity analyses	9-10
**Discussion**			
Key results	18	Summarise key results with reference to study objectives	11
Limitations	19	Discuss limitations of the study, taking into account sources of potential bias or imprecision. Discuss both direction and magnitude of any potential bias	12
Interpretation	20	Give a cautious overall interpretation of results considering objectives, limitations, multiplicity of analyses, results from similar studies, and other relevant evidence	12
Generalisability	21	Discuss the generalisability (external validity) of the study results	12
**Other information**			
Funding	22	Give the source of funding and the role of the funders for the present study and, if applicable, for the original study on which the present article is based	13

*Give information separately for exposed and unexposed groups.

Note: An Explanation and Elaboration article discusses each checklist item and gives methodological background and published examples of transparent reporting. The STROBE checklist is best used in conjunction with this article (freely available on the Web sites of PLoS Medicine at http://www.plosmedicine.org/, Annals of Internal Medicine at http://www.annals.org/, and Epidemiology at http://www.epidem.com/). Information on the STROBE Initiative is available at http://www.strobe-statement.org.

Supplementary File S2: Data extraction tool

Variable	Variable explanation and options
No	List the numbers in chronological order
Sex	M/F (as listed in the medical record)
Residential postcode	4 digits
Cancer Type	Lung CA<br>Liver CA
Year of Cancer Diagnosis	DD/MM/YYYY
Additional diagnosis	List all other additional diagnosis
Date of first episode of care	First episode of care is defined as 'the date when the patient was first seen by a healthcare practitioner for management, consultation or treatment of the cancer'.
Age at the first episode of care	Write the age as recorded when the first episode of care occurred.
Date of death	List date of death if available or recorded,
Place of death	Acute care/Hospital, Hospice/Palliative care, Home, Nursing home, Other
Preferred place of death	Note the patient's preferred place of death as documented in the records. If not available state 'Not documented'.
**Socio-demographic measurements**	
Country of birth	Country of birth as documented in the patient record
Preferred language	English, Non-English, Not Specified
Language spoken as home	English, Non-English, Not Specified
Interpreter required	Yes, No or Not specified
Religion	As documented in the patient record
Year of arrival in Australia	As documented in the patient record
Parents country of birth (if available)	As documented in the patient record
Refugee status	As documented in the patient record
**Potential burdensome end-of-life care measurements**	
Was there a documentation of ≥ 14 days in hospital in the last 30 days of life (excluding palliative or hospice care)	Yes, No or NA
Was there a documentation of ≥ 3 hospitalisations in the last 90 days of life (excluding palliative or hospice care)	Yes, No or NA
Was there a documentation of last does of IV chemotherapy in the last 14 days of life	Yes, No or NA
Was there a documentation of new chemotherapy treatment commenced in last 30 days of life	Yes, No or NA
Was there a documentation of Radiotherapy in last 30 days of life	Yes, No or NA

Was there a documentation of > 1 ED visit in last 30 days of life	Yes, No or NA
Was there a documentation of >1 hospitalisation in last 30 days of life	Yes, No or NA
Was there a documentation of admission to ICU in last 30 days of life	Yes, No or NA
Was there documentation of resuscitation attempted in last 30 days of life?	Yes, No or NA
Was there a documentation of Mechanical ventilation in last 30 days of life	Yes, No or NA
Was there a documentation of Place of death in Acute care (excluding palliative or hospice care)	Yes, No or NA
**ACP communication measurements**	
Any ACP Communication	Yes or No
Date of ACP Communication	Write the date when the communication occurred or recorded in the medical records
Communication documentation type	Formal<br>- CPR forms/Acute resuscitation forms<br>- Advance care directive<br>- Advance care plan<br>- Ambulance care plan<br>- PEG feed directive<br>- Patients' expectations or goals of care form<br>- Mechanical ventilation form/plan<br>- Hospital admission/re-admission plan<br>- Personal wills<br>- Substitute decision-maker form<br>- Directive for any other procedure or treatment<br>- Other<br><br>Informal<br>- Communication about formal documents<br>- Communication about place of death or end-of-life care<br>- Communication about future treatment planning/directives<br>- Communication about personal wishes and goals<br>- Communication about prognosis/life expectancy<br>- Communication about concerns and hopes towards future<br>- Communication about cultural or religious preferences<br>- Communication about end-of-life, death, palliative care<br>- Other
Setting	Did the communication/documentation occur when the patient was<br>- Inpatient/hospital<br>- ED<br>- Outpatient<br>- Community<br>- Hospice/Palliative care<br>- Other
**Palliative care measurements**	

Referral made to palliative care team	Yes or No
Seen by palliative care team/staff	Yes or No
Any other important notes	Write any other noteworthy information
